# Timing of continuous renal replacement therapies in acute kidney injury patients after cardiac surgery

**DOI:** 10.1186/2197-425X-3-S1-A261

**Published:** 2015-10-01

**Authors:** P Cardenas Campos, J Sabater Riera, G Moreno Gonzalez, VF Corral Velez, JM Vazquez Reveron, RS Contreras Medina, ND Toapanta Gaibor, VD Gumucio Sanguino, XL Perez Fernandez

**Affiliations:** Hospital Universitari de Bellvitge, Barcelona, Spain

## Introduction

Cardiac surgery-associated acute kidney injury (CSA-AKI) is a common postoperative complication, despite advances in surgical techniques and intensive care, mortality and morbidity associated with acute renal failure have not markedly changed in the last decade. Depending on the definition used, the incidence of CSA-AKI is reported to range from 1 to 30%. CSA-AKI requiring continuous renal replacement therapy (CRRT) occurs in 1.2-3.0% of cardiac surgery cohorts and is independently associated with mortality.

## Objectives

We studied mortality differences between CRRT and nonCRRT patients. We studied Timing effect (Early vs Late CRRT initiation respect to ICU admission) using non-CRRT as control group.

## Methods

We performed an observational prospective cohort study of 2100 patients undergoing different types of cardiac surgery, from 2006 to 2011 in a tertiary university hospital. Of these, 87 patients developed AKIN 2-3 stage within the first 24h of immediate postsurgical ICU admission, whereas 30 patients received CRRT during their ICU stay. All those patients with CKD 4-5 were excluded. Severity of AKI was defined by AKIN score (urine output (UO) and serum creatinine). We classified patients in early group ( < 48 hours) and late group (>48 hours) based on time from ICU admission to CRRT.

## Results

Baseline patients characteristics were: Mean age (years) 65 ± 10; APACHE II 20 ± 8; SOFA 10 ± 3; UO (ml) 6h before CRRT 161 ± 225; Creatinine at CRRT initiation (umol/L) 298,20 ± 98. Mortality at 90 days in patients with AKIN 2-3 the first 24h of ICU admission was 37,9%. The mortality in patients with AKIN 2-3 that required CRRT was 63,3%, compared with 24,6% in patients that did not require CRRT. Timing analysis was performed (Early vs Late) using non CRRT as control group: Mean age in years (60 ± 8 vs 70 ± 7) vs 66 ± 10; SOFA (11 ± 3 vs 11 ± 3) vs 10 ± 3; APACHE (22 ± 6 vs 23 ± 7) vs 19 ± 8; Creatinine at ICU admission (umol/L) (55 ± 14 vs 66 ± 17) vs 59 ± 8; UO 24h at ICU admission (ml/k/h) (0,3 ± 0,2 vs 0,7 ± 0,5) vs 0,8 ± 0,4; Urine output 6h previous to CRRT (0,2 ± 0,2 vs 0,5 ± 0,6)*. *p < 0,05.

## Conclusions

Higher mortality in those AKIN 2-3 whom needed CRRT vs non-CRRT (63.3% vs 24.6%). Timing analysis revealed no survival differences between those patients who were early initiated on CRRT ( < 48 h respect to ICU admission) and those who were initiated later (60.0% VS 66.7%). Urine output 6h previous to CRRT was significative higher in the late group respect to the early group (0.54 ml/kg/h vs 0.18 ml/gh/h) introducing some concerns about CRRT indication on this Late group. CRRT initiation in advanced AKIN patients (AKIN 2-3) who still present preserved UO > 0.5 ml/k/h should be discouraged, these patients present higher mortality compared to non-CRRT patients.

